# Patient Preferences for In-Person vs Remote Care for Long-Term Conditions

**DOI:** 10.1001/jamanetworkopen.2025.57759

**Published:** 2026-02-10

**Authors:** Tiphaine Lenfant, Elodie Perrodeau, Philippe Ravaud, Viet-Thi Tran

**Affiliations:** 1 Center for Research in Epidemiology and Statistics, Université Paris Cité and Université Sorbonne Paris Nord, Institut National de la Santé et de la Recherche Médicale, Institut National de la Recherche pour l’Agriculture, l’Alimentation et l’Environnement, Paris, France; 2 Médecine Interne, Assistance Publique–Hôpitaux de Paris (APHP), Hôpital Européen Georges Pompidou, Paris, France; 3 Centre d’Épidémiologie Clinique, APHP, Hôpital Hôtel-Dieu, Paris, France; 4 Department of Epidemiology, Columbia University Mailman School of Public Health, New York, New York

## Abstract

**Importance:**

As health care systems face greater strain due to the increasing prevalence of long-term conditions (LTCs) and workforce shortages, optimizing remote care could help maintain access to timely follow-up care while alleviating pressure on in-person services.

**Objectives:**

To assess the availability and use of 3 remote modalities by patients with LTCs for interacting with their referring physician; their preference for in-person vs remote consultation for different situations; and their willingness to trade an in-person consultation with their referring physician in 20 days for an earlier remote consultation with a nonreferring physician.

**Design, Setting, and Participants:**

This survey study was conducted from April 1 to August 28, 2024, in France. Adult patients with at least 1 LTC participating in a nationwide e-cohort were recruited to complete an online survey. To obtain estimates representative of patients with LTCs in France, analyses were performed on a weighted dataset with calibration on margins from the 2021 Santé Publique France Survey.

**Exposures:**

Each patient was presented with 1 of 5 randomly assigned situations regarding worsening symptoms, new symptoms, annual checkup, medication renewal, and results discussion.

**Main Outcomes and Measures:**

Responses were assessed for (1) availability and use of remote modalities (video consultation, telephone contact, and asynchronous message exchange) for interacting with the referring physician; (2) patient preference for in-person or remote modalities when interacting with the referring physician depending on the situation; and (3) patient willingness to trade an in-person consultation with their physician in 20 days for an earlier direct-to-consumer (DTC) remote consultation with a nonreferring physician.

**Results:**

Among 1995 participants (mean [SD] age, 55 [17] years; 56% women), 47% reported that no remote modalities for communicating with their referring physician were available. (All percentages presented are weighted.) Up to 55% preferred remote over in-person consultation to interact with their referring physician, with higher proportions for results discussion (43%) and medication renewal (55%) than for worsening symptoms (36%), new symptoms (25%), and annual checkup (26%). Up to 51% of patients were willing to trade an in-person consultation with their referring physician in 20 days for a DTC remote consultation within 5 days with a nonreferring physician.

**Conclusions and Relevance:**

This survey study showed that patients with LTCs had limited access to remote modalities to interact with their referring physician for follow-up care, and patients considered replacing an in-person with a remote consultation even with another physician depending on the situation and the length of delay. Further research is needed to investigate the effective integration of remote care into the health care system in ways that maintain patient-centered care and improve patient outcomes.

## Key Points

**Question:**

How and when would patients with long-term conditions (LTCs) be willing to trade in-person consultations for remote care with their referring physician and with a nonreferring physician?

**Findings:**

In this survey study of 1995 adults with LTCs, remote modalities for interacting with their referring physician were not available to all participants. Depending on the situation, 25% to 55% of patients preferred remote over in-person consultation with their referring physician and 20% to 51% were willing to trade an in-person consultation in 20 days with their referring physician over an earlier remote consultation with a nonreferring physician, especially for new or worsening symptoms.

**Meaning:**

The findings of this study suggest that care models should be redesigned to offer context-appropriate blended care options to adults with LTCs.

## Teaser

This survey study examines the availability, use, and preference for video, telephone, and messaging contact with physicians among individuals with chronic disease in France.

## Introduction

One-third of adults in Western countries have long-term conditions (LTCs).^1,2,3^ The follow-up of LTC, traditionally conducted during in-person consultations in current health care systems, imposes several logistical and emotional burdens on patients.^4^ First, patients must make personal and professional arrangements to attend in-person consultations, which may involve long travel and waiting times. For example, in Germany, a study found that patients needed to allocate over 1 hour for a 13-minute consultation with a neurologist.^5^ Second, consultations are prescheduled and ill-timed as patients see their physician at the scheduled time rather than when the care is needed. For example, guidelines for rheumatoid arthritis recommend consultations every 3 to 6 months, which is not adapted either for the majority of patients who are stable or for the minority of patients who may have a flareup between visits that still could be missed and left untreated.^6^ Third, between consultations, patients are left alone to manage their conditions, with limited guidance on when and how to seek help.^7^ All of these burdens are amplified for patients with multiple LTCs, who represent approximately 50% of patients with LTCs and who must deal with these issues across different physicians and practices.^1,2,3,8,9^

Remote care could address some of these burdens by reducing travel time and facilitating communication at the right moment.^4,10,11^ Remote care can be either synchronous (via video consultations or telephone contacts) or asynchronous (via messaging on secure health platforms or patient portals). Synchronous modalities mimic the dynamics of an in-person consultation by enabling real-time interaction between the patient and the physician while alleviating geographic and personal constraints.^12,13^ Asynchronous modalities enable patients to send information, ask questions, or share documents with their physicians at any time, without the fear of disturbing and waiting for availability, as responses are not expected in real time.^14,15^ Beyond communication modalities, remote care can be delivered by the referring physician or by a nonreferring physician through direct-to-consumer (DTC) remote consultations.^16^

In the literature, studies report conflicting results on patients’ preferences about remote care. On one hand, when asked about a single consultation, patients often indicated their preference for in-person care, which allows for human contact and physical examination.^13,17,18,19^ On the other hand, when asked about how they envision their long-term treatment, 31% of patients indicated that video consultations could replace in-person contacts for more than 50% of their consultations.^10^ Moreover, few studies have investigated preferences for remote care based on specific clinical contexts, such as stable vs unstable LTCs, medication renewals, or discussions of results.^10,15,20^ It is unclear whether patients would be willing to use DTC remote consultations when their referring physician is unavailable within an acceptable time frame.^16^

We thus aimed to evaluate, among patients with LTCs in France, (1) the availability and use of 3 remote modalities for interacting with their referring physician, (2) their preference for an in-person or remote consultation with their referring physician regarding 1 of 5 situations, (3) their willingness to trade an in-person consultation with their referring physician in 20 days for an earlier remote consultation with a nonreferring physician.

## Methods

From April 1 to August 28, 2024, we conducted an online vignette-based survey to assess patient preferences regarding follow-up care for their LTCs. Vignettes are descriptions of hypothetical scenarios accompanied by questions to elicit participants’ preferences.^21,22^ Responses to vignette-based surveys have been shown to adequately represent actual behaviors, including in situations with high desirability bias.^21,22^ The CER Université Paris Cité approved this survey study. All participants provided informed consent electronically after reviewing related information. We followed the Checklist for Reporting Results of Internet E-Surveys (CHERRIES) reporting guideline.^23^

### Participants

We recruited adults aged 18 years or older with any LTC—defined as a health condition requiring care for more than 6 months—from the Community of Patients for Research (ComPaRe), a nationwide e-cohort of more than 60 000 individuals with chronic conditions in France.^24^ ComPaRe uses a multimodal recruitment strategy (outreach through hospitals, medical societies, social media, mass media, and patient networks) that enables the inclusion of participants from primary care, mental health care, and medical and surgical specialties, with diversity in terms of socioeconomic background and access to care.^25^ Patients who had been active in the e-cohort within the 12 months preceding the study and who had consented to be invited to external studies were recruited to participate.

### Survey Design and Administration

Participants were invited to complete an online survey structured into 3 parts. First, the survey assessed the availability and use of 3 remote modalities (video consultation, telephone contact, and asynchronous message exchange [AME]) for patient communication with their referring physician (ie, the physician who provided the most regular follow-up care for the LTC). Demographic characteristics, health data, and referring physician information were also gathered (eMethods 1 in Supplement 1).

Second, the survey assessed patients’ preferences between an in-person and a remote consultation (using 1 of the 3 remote modalities) with their referring physician by presenting each participant with 1 of 5 randomly assigned situations or vignettes regarding worsening symptoms, new symptoms, annual checkup, medication renewal, and results discussion. Each vignette started with “Imagine the following situation:” and was accompanied by a description. Vignette 1 posed, “You are experiencing a worsening of your usual symptoms. You know these symptoms because you have had them in the past.” In vignette 2, “You have a new symptom. It is a symptom you have never had before. You do not think it requires an emergency visit.” In vignette 3, “You need an annual check-up on your long-term conditions. Your symptoms are stable.” In vignette 4, “You need to renew the prescription for your long-term treatment. Your symptoms are stable.” In vignette 5, “You have received test results for your long-term condition(s). You have not noticed anything alarming in the results.” The vignettes ended with “You would like to consult your referring physician.” Patients were presented with 4 response options: in-person consultation, video consultation, telephone contact, or AME.

Third, the survey assessed patients’ willingness to trade an in-person consultation in 20 days with their referring physician for an earlier DTC remote consultation with a nonreferring physician (ie, an unknown physician, other than the patient’s usual physician, able to provide follow-up care for an LTC) regarding the same situation or vignette. It asked, “In this situation, imagine you could see your referring physician in person in 20 days, would you rather see an unknown physician that could see you remotely sooner?” Response options were as follows: (1) “Yes, I would like to see this unknown physician remotely sooner”; (2) “It depends on the delay to this remote consultation with the unknown physician”; or (3) “No, I would rather see my referring physician in person in 20 days.” If participants opted for “It depends on the delay,” they were provided with 4 successive delay reductions—“What if the remote appointment with this unknown physician was in 15 to 20 days,” “in 10 to 15 days,” “in 5 to 10 days,” or “within 5 days”—and could opt for the DTC consultation during this delay or wait for the later in-person consultation. The DTC remote option with another physician was always presented with a shorter delay than the fixed 20-day in-person consultation with the referring physician, and the delay was progressively reduced to assess how willingness to trade changed as the time advantage increased.

The survey was tested in a pilot phase of 30 patients with LTCs (eMethods 2 in Supplement 1). It was available online on PROCESS (Platform for Research Online and CitizEn Science Surveys). Participation was anonymous.

### Statistical Analysis

To obtain estimates that were representative of the population of patients with LTCs in France, we applied calibration on margins, a reweighting procedure that adjusts survey weights so that the marginal distributions of the sample match known population totals for selected auxiliary variables.^26^ We used the 2021 Santé Publique France Survey to obtain age categories, gender, and educational level distributions as auxiliary variables. Descriptive results are presented on the weighted dataset.

We described the availability and use of video consultation, telephone contact, and AME for interaction between patients and their referring physician. Additionally, we described the proportion of patients preferring remote over in-person consultation with their referring physician for each of the 5 situations presented. The 3 remote modalities (video consultation, telephone contact, and AME) were considered separately and pooled into a single remote variable. We conducted a logistic regression model to identify the factors associated with patient preferences for remote care. The dependent variable was the preference (in-person vs remote consultation), and odds ratios (ORs) higher than 1 indicated a higher likelihood of preferring remote care. Covariates were patient, physician, and consultation characteristics (eMethods 3 in Supplement 1). We conducted a predefined subgroup analysis among participants who had never used any of the 3 remote modalities to interact with their physician.

We described the proportion of patients who were willing to trade a later in-person consultation with their referring physician for an earlier DTC remote consultation with a nonreferring physician, in each of the 5 situations and for each delay reduction. We conducted a logistic regression model to identify factors associated with this preference. The dependent variable was the willingness pooled in a binary variable: no (“No, I would rather see my referring physician in person in 20 days”) vs yes or open (“It depends on the delay” or “Yes, I would like to see this unknown physician remotely sooner”). ORs higher than 1 indicated a higher likelihood of willingness to trade later in-person care for an earlier DTC remote consultation. Covariates were patient, physician, and consultation characteristics (eMethods 3 in Supplement 1).

Statistical significance was defined as a 2-sided *P* < .05. Analyses were conducted using R, version 4.3.0 (R Project for Statistical Computing), and the ICARUS package.^27^

## Results

Among the 2328 patients who consented to participate in the study, 2038 completed the survey. A total of 1995 patients who had sufficient data for computation of the calibration of margin were analyzed (weighted population) (eFigure 1 in Supplement 1). All percentages presented are weighted.

After weighting, the patient population had a mean (SD) age of 55 (17) years, of whom 56% were women and 69% had multimorbidity. The most reported conditions were rheumatologic diseases (31%), high blood pressure (30%), and mental health disorders (27%). Most of the referring physicians (68%) were general practitioners (GPs). The delay between departure from home or work and the start of an in-person consultation was 1 hour or more for 37% of patients. The proportion of participants living in zones with high or low GP density was similar to estimates in the general population. In total, 79% of patients reported it easy to free up time (half a day or more) for an in-person consultation (Table; eTable 1 in Supplement 1).

**Table.** Characteristics of Patients, Physicians, and Consultations

Characteristics	Dataset, No. (%)	
	Raw (n = 1995)	Weighted %^a^
Patients		
Age, mean (SD), y	52 (15)	55 (17)
Gender		
Man	562 (28)	44
Woman	1433 (72)	56
Highest educational level		
>Bachelor’s degree	1209 (61)	17
≤Bachelor’s degree	786 (39)	83
Self-perception of financial situation		
Difficult	697 (35)	39
Not difficult	1298 (65)	61
Multimorbidity: ≥2 LTCs	1323 (66)	69
Self-assessed anxiety level^b^		
≤4 of 6	1201 (60)	59
>4 of 6	794 (40)	41
Self-management score^c^		
Good to excellent	1248 (63)	59
Poor to medium	747 (37)	41
DHCLS score^d^		
Higher: >10 of 15	1729 (87)	83
Lower: ≤10 of 15	266 (13)	17
Reported LTC		
Rheumatologic diseases	558 (28)	31
Mental health disorders	530 (27)	27
High blood pressure	439 (22)	30
Gynecological diseases	373 (19)	12
Endocrine diseases	295 (15)	14
Hepatic and gastroenterological diseases	294 (15)	15
Dermatologic diseases	277 (14)	16
Inflammatory and immune-mediated diseases	233 (12)	12
Neurologic diseases	229 (12)	13
Pulmonary diseases	221 (11)	13
Diabetes	203 (10)	15
Ophthalmologic diseases	193 (10)	12
Cardiac diseases	179 (9)	12
Hematologic and oncologic diseases	161 (8)	9
Kidney diseases	108 (5)	6
Infectious diseases	46 (2)	2
Other^e^	525 (26)	26
Referring physicians		
Physician specialty		
General practitioner	1259 (63)	68
Specialist	735 (37)	32
Missing data	1 (<1)	0
Length of patient relationship with physician, y		
≥5	1167 (58)	59
<5	821 (41)	41
Missing data	7 (<1)	<1
Listening skills evaluated by patient		
Good to excellent	1553 (78)	77
Poor to medium	440 (22)	23
Missing data	2 (<1)	0
Consultations		
Time from departure to start of in-person consultation, h		
<1	1195 (60)	63
≥1	795 (40)	37
Missing data	5 (<1)	<1
Delay before the next available in-person consultation, wk		
<2	1018 (51)	56
≥2	970 (49)	44
Missing data	7 (<1)	<1
Ability to make personal or professional arrangements to free up time (half-day or entire day) to attend in-person consultation		
Difficult to impossible	564 (28)	21
Easy	1429 (72)	79
Missing data	2 (<1)	<1
Most frequently used modality		
In-person consultation	1856 (93)	94
Telephone contact	12 (1)	<1
Video consultation	102 (5)	5
AME	24 (1)	1
Missing data	4 (<1)	<1
Availability and use of remote modalities		
Not available or never used (unserved)	857 (43)	47
Used	1137 (57)	53
Missing data	1 (<1)	0

Abbreviations: AME, asynchronous message exchange; DHCLS, Digital Health Care Literacy Scale; LTC, long-term condition.

^a^ All percentages are weighted proportions derived from the calibrated dataset.

^b^ Anxiety level range: 1 to 6, with 6 indicating highest severity.

^c^ Self-management score range: 1 to 6, with 6 indicating best self-management.

^d^ DHCLS score range: 3 to 15, with 15 indicating highest digital literacy.

^e^ Other was an option the patient could select if their LTC category was not included.

### Availability and Use of Remote Modalities (n = 1994)

**Figure 1.**
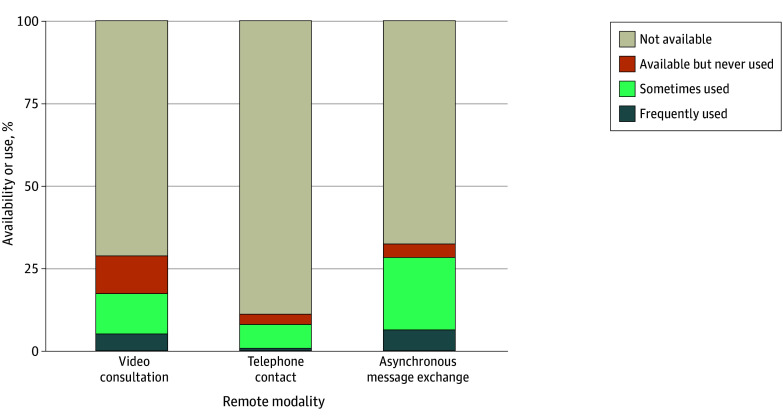
Component Bar Graph of the Availability and Use of Remote Modalities for Patient Interaction With the Referring Physician The referring physician is defined as the physician providing the most regular follow-up care for the patient’s long-term conditions. Weighted dataset includes 1994 patients.

To interact with their referring physician, video consultations were available to 28% of patients, telephone contacts to 11% of patients, and AMEs to 32% of patients (Figure 1). For 47% of patients, none of these 3 remote modalities was available for use with the referring physician, rendering these patients unserved.

### Patients’ Preferences for In-Person vs Remote Consultations With Their Referring Physician (n = 1908)

**Figure 2.**
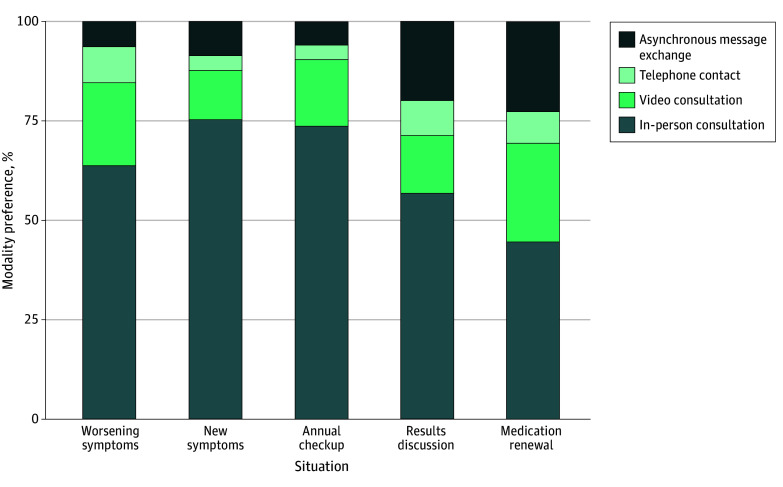
Component Bar Graph of Patient Preference for In-Person Consultation or Remote Modalities for Interacting With the Referring Physician in 5 Situations The referring physician is defined as the physician providing regular follow-up care for the patient’s long-term conditions. Weighted dataset includes 1908 patients.

Across the 5 situations, a remote modality was preferred by 37% of patients. Synchronous mediums (video consultation and telephone contact) were preferred over AMEs for all 5 situations (eg, 33% vs 22% for medication renewal; 30% vs 6% for worsening symptoms). This preference for remote care was higher for results discussion and medication renewal (43% and 55%) than for worsening symptoms, new symptoms, and annual checkup (36%, 25%, and 26%) (Figure 2). These results were similar among the 47% of unserved patients, with 36% preferring an in-person consultation in their situation (eFigure 2 in Supplement 1).

Adjusted analyses identified factors significantly associated with the preference for remote care (eTable 2 in Supplement 1). Being unserved was not associated with the preference for in-person over remote consultation (OR, 1.06; 95% CI, 0.86-1.30; *P* = .58). Three situations were associated with the preference for in-person care: worsening symptoms (OR, 0.39; 95% CI, 0.29-0.53; *P* < .001), new symptoms (OR, 0.28; 95% CI, 0.21-0.38; *P* < .001), and annual checkup (OR, 0.27; 95% CI, 0.20-0.37; *P* < .001), with medication renewal as the reference situation. Patient characteristics such as man gender (OR, 0.73; 95% CI, 0.57-0.92; *P* = .008), lower digital health literacy score (OR, 0.51; 95% CI, 0.37-0.70; *P* < .001), and easy ability to free up time (OR, 0.47; 95% CI, 0.37-0.60; *P* < .001) were associated with the preference for in-person consultation. Physician characteristics such as medium-level or poor listening skills (OR, 1.35; 95% CI, 1.06-1.73; *P* = .02) and longer delay before the next in-person appointments (OR, 1.38; 95% CI, 1.09-1.74; *P* = .007) were associated with the preference for remote consultation. The subgroup analysis among unserved patients yielded similar results (eTable 3 in Supplement 1).

### Patients’ Willingness to Trade In-Person Consultation With Their Referring Physician for Earlier DTC Remote Consultation With a Nonreferring Physician (n = 1940)

**Figure 3.**
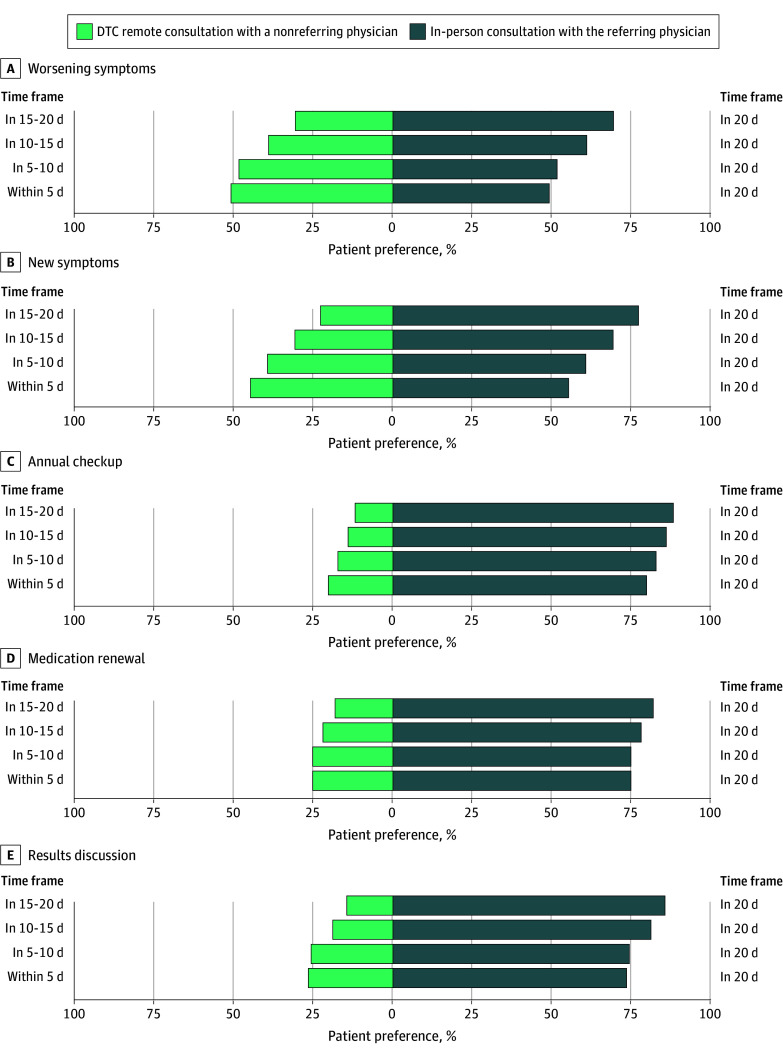
Tornado Plot Demonstrating Patient Willingness to Trade In-Person Consultation With the Referring Physician for Earlier Direct-to-Consumer (DTC) Remote Consultation With a Nonreferring Physician in 5 Situations The referring physician is defined as the physician providing regular follow-up care for the patient’s long-term condition. For example of this willingness to trade, consider the worsening symptoms situation in which 31% of patients prefer a DTC remote consultation occurring in 15 to 20 days rather than an in-person consultation with their referring physician occurring in 20 days. The sooner the remote consultation can be scheduled, the higher this preference proportion grows. Weighted dataset includes 1940 patients.

eTable 4 in Supplement 1 and Figure 3 present patients’ willingness to trade an in-person consultation with their referring physician in 20 days for an earlier DTC remote consultation with a nonreferring physician. The proportion of patients being willing to trade for the remote option increased as the delay shortened. Willingness also varied by situation. Patients were more likely to choose remote consultation for worsening or new symptoms than for annual checkup, medication renewal, or results discussion. When confronted with worsening symptoms, 51% of patients preferred a DTC remote consultation within 5 days with another physician rather than wait 20 days to consult their referring physician in person. In contrast, for their annual checkup, 20% of participants preferred the DTC remote consultation within 5 days rather than wait 20 days for an in-person consultation with their referring physician.

Adjusted analyses identified factors significantly associated with the preference for an earlier DTC remote care (eTable 5 in Supplement 1). Clinical situations such as new symptoms (OR, 3.16; 95% CI, 2.35-4.26; *P* < .001) and worsening symptoms (OR, 3.74; 95% CI, 2.77-5.08; *P* < .001) were associated with the preference for an earlier DTC remote consultation (with medication renewal as the reference situation). Patient characteristics such as man gender (OR, 1.32; 95% CI, 1.04-1.67; *P* = .02), difficulty with freeing up time for an in-person consultation (OR, 1.54; 95% CI, 1.22-1.96; *P* < .001), and no available remote modalities for interacting with the referring physician (OR, 1.33; 95% CI, 1.09-1.64; *P* = .006) were associated with the preference for an earlier remote consultation. Physician characteristics such as a GP specialty (OR, 1.79; 95% CI, 1.28-2.50; *P* = .001), medium-level or poor listening skills (OR, 1.53; 95% CI, 1.20-1.95; *P* = .001), and usual delay of fewer than 2 weeks (OR, 1.43; 95% CI, 1.12-1.82; *P* = .003) were associated with the preference for an earlier DTC remote consultation.

## Discussion

This nationwide vignette-based survey study found that, for 47% of participants, none of the 3 remote modalities (video consultation, telephone contact, or AME) was available for interacting with their referring physician. Results of this study are consistent with national statistics, which show that, in France in 2023, only 4% of consultations were video consultations, a figure lower than in other Western countries, such as Spain (29%) and Denmark (31%).^28^ Although remote care is expected to improve access for patients with mobility limitations and geographic barriers, its current use in France is skewed toward younger, urban populations, with more than half of all video consultations taking place in the Paris region and less than 20% in rural areas (representing a third of the French population).^28^ Another issue regarding the availability and use of remote care is that some patients were unaware of whether remote modalities were offered by their referring physician.^20^ In 2023, GPs conducted 229 million in-person consultations.^28^ Based on French data, we assumed that the 5 situations we presented reflected approximately 58% of GP consultations (the remaining 42% were acute, urgent, preventive, administrative, pediatric, or pregnancy-related consultations).^29^ In our study, 37% of patients preferred to use remote modalities in their assigned situation. A macro-level extrapolation suggests that more than 40 million in-person GP consultations could be shifted to remote care if patient preferences were followed. This estimate is purely illustrative and should not be interpreted as a recommendation, given that decisions about the mode of consultation must remain shared between physicians and patients and depend on the clinical context (eMethods 4 in Supplement 1).

Our findings suggest that up to half of patients would be willing to trade in-person consultations for remote consultations in certain situations, especially when they anticipate that no physical examination or complex decision-making is needed.^10,13,17,20,30^ In these situations, remote care may not only be sufficient but also preferable, especially when it helps reduce the burden of travel, waiting time, and time away from work or family. Video consultation and telephone contact were preferred over AMEs for all 5 situations. These results contrast with data from a primary care study in Canada, where 82% (out of 13 174) of virtual visits were requested via AME rather than by video consultation or telephone contact.^15^ One reason that may explain this difference is that secured AME in France was launched at the national level only in 2022.^31^ Furthermore, lower DHCLS score was associated with a greater preference for in-person consultations, consistent with previous studies.^32^ These results confirm that care delivery should be adapted to fit patient preferences but also possibilities, without imposing digital tools indiscriminately on all patients.^33^

Our study highlighted patients’ willingness to trade for DTC remote consultations when situations felt more urgent, such as the occurrence of new symptoms or worsening of existing symptoms. In these situations, up to 51% of participants preferred earlier DTC remote consultations. This choice resulted in a loss of continuity of care since the physician providing care was not the referring physician and lacked access to the medical record. This willingness to trade continuity for speed may put patients at risk, as continuity of care is associated with better health outcomes.^34^ A Canadian study found that patients who underwent a DTC remote consultation were 3 times more likely to visit the emergency department within 7 days than patients who saw their referring physician.^35^ These findings caution against overreliance on DTC remote models without ensuring adequate integration into health care systems.

Overall, our findings suggest the need to rethink the organization of follow-up care for patients with LTCs. Remote care is underused, not because patients reject it but because it may not be sufficiently offered, accessible, or adapted to patient needs. Remote care should not be added as a parallel layer to replace in-person care; rather, it should be thoughtfully integrated as a blended care model, where in-person and remote modalities are complementary and coordinated. This approach could reduce unnecessary consultations and reserve in-person care for situations where it brings the most value.^36^

### Strengths and Limitations

This study has several strengths. First, it was a large nationwide study that included participants with diverse LTCs recruited from a French e-cohort, which was weighted to approximate the French population of patients living with LTCs. Second, we conducted a vignette-based survey, a methodologically robust tool that allowed us to capture nuanced, context-dependent preferences that might be missed in a single generic survey on remote care.

This study has several limitations as well. First, our sample was not a representation of patients currently using remote care, but rather it presented the views of patients with LTCs in general. We decided to explore the general preferences of these patients, for whom remote care may likely be expanded in the future. Second, the sample was a group of volunteers with internet access and high DHCLS score, recruited via an e-cohort. These participants were younger, had higher educational levels, and were more digitally skilled than the national population of patients with LTCs. The conclusions may not reflect the implications of greater remote care for the wider population with LTCs. Despite weighting, selection bias is possible; however, patients with less digital literacy were still represented, with lower DHCLS score (Table). Third, the response rate was relatively low compared with the number of invited participants, but this discrepancy reflects the voluntary nature of participation and the fact that patients were invited to get involved in a study outside of their usual cohort.

## Conclusions

In this study, patients with LTCs were willing to incorporate remote consultation into their follow-up care, particularly when the modality aligned with the clinical context and personal constraints. This volunteer sample had access to internet and had high digital literacy. The conclusions may not reflect the implication of greater remote care for the wider population with LTCs. Further research on blended care should investigate how to effectively integrate remote care into the health care system in ways that maintain patient-centered care and improve patient outcomes.

## Notes

Supplement 1. **eMethods 1.** Data Collection **eMethods 2.** Pilot Phase **eMethods 3.** Covariates of the Logistic Regression Models **eMethods 4.** Number of In-Person GP Consultations That Could Be Replaced by Remote Care in France **eTable 1.** Patients, Physicians and Consultations Characteristics Described by Clinical Situation (n=1995) **eTable 2.** Factors Associated With Patients’ Preferences for Remote Modalities With Their Referring Physician for the Follow-up of Their Long-Term Conditions (Logistic Regression Model) **eTable 3.** Factors Associated With the Preference for a Remote Modality Over an In-Person Consultation With the Referring Physician Among Unserved Patients (Patients With No Access to Any of the Three Remote Modalities to Interact With Their Physician), Logistic Regression Model **eTable 4.** Patients’ Willingness to Trade for a Quicker DTC Remote Consultation With a Non-Referring Physician Rather Than Waiting 20 Days to Consult Their Referring Physician in Person, Depending on the Delay Before the DTC and the Clinical Situation (Weighted Data Set, n=1940) **eTable 5.** Factors Associated With Patients’ Preferences for a Quicker Direct-to-Consumer Remote Consultation With a Non-Referring Physician Rather Than an In-Person Consultation With Their Referring Physician in 20 Days, (Logistic Regression Model) **eFigure 1.** Flow Chart **eFigure 2.** Preferences for In-Person Consultation or Remote Modalities With the Referring Physician, Depending on the Clinical Situation (Weighted Data Set, n=1908) of All Patients (Panel A) and of Unserved Patients (Panel B)

Supplement 2.
Data Sharing Statement
