# Supplementary material for: Patient Preferences for In-Person vs Remote Care for Long-Term Conditions
Source: JAMA Netw Open. 2026 Feb 10;9(2):e2557759. doi: 10.1001/jamanetworkopen.2025.57759 (PMC12892154; doi:10.1001/jamanetworkopen.2025.57759)
Supplement: Supplement 1. — eMethods 1. Data Collection eMethods 2. Pilot Phase eMethods 3. Covariates of the Logistic Regression Models eMethods 4. Number of In-Person GP Consultations That Could Be Replaced by Remote Care in France eTable 1. Patients, Physicians and Consultations Characteristics Described by Clinical Situation (n=1995) eTable 2. Factors Associated With Patients’ Preferences for Remote Modalities With Their Referring Physician for the Follow-up of Their Long-Term Conditions (Logistic Regression Model) eTable 3. Factors Associated With the Preference for a Remote Modality Over an In-Person Consultation With the Referring Physician Among Unserved Patients (Patients With No Access to Any of the Three Remote Modalities to Interact With Their Physician), Logistic Regression Model eTable 4. Patients’ Willingness to Trade for a Quicker DTC Remote Consultation With a Non-Referring Physician Rather Than Waiting 20 Days to Consult Their Referring Physician in Person, Depending on the Delay Before the DTC and the Clinical Situation (Weighted Data Set, n=1940) eTable 5. Factors Associated With Patients’ Preferences for a Quicker Direct-to-Consumer Remote Consultation With a Non-Referring Physician Rather Than an In-Person Consultation With Their Referring Physician in 20 Days, (Logistic Regression Model) eFigure 1. Flow Chart eFigure 2. Preferences for In-Person Consultation or Remote Modalities With the Referring Physician, Depending on the Clinical Situation (Weighted Data Set, n=1908) of All Patients (Panel A) and of Unserved Patients (Panel B) [file jamanetwopen-e2557759-s001.pdf]

Supplemental Online Content

Lenfant T, Perrodeau E, Ravaud P, Tran VT. Patient preferences for in-person vs remote care for long-term conditions. *JAMA Netw Open*. 2026;9(2):e2557759. doi:10.1001/jamanetworkopen.2025.57759

eMethods 1. Data Collection

eMethods 2. Pilot Phase

eMethods 3. Covariates of the Logistic Regression Models

eMethods 4. Number of In-Person GP Consultations That Could Be Replaced by Remote Care in France

eTable 1. Patients, Physicians and Consultations Characteristics Described by Clinical Situation (n=1995)

eTable 2. Factors Associated With Patients' Preferences for Remote Modalities With Their Referring Physician for the Follow-up of Their Long-Term Conditions (Logistic Regression Model)

eTable 3. Factors Associated With the Preference for a Remote Modality Over an In-Person Consultation With the Referring Physician Among Unserved Patients (Patients With No Access to Any of the Three Remote Modalities to Interact With Their Physician), Logistic Regression Model

eTable 4. Patients' Willingness to Trade for a Quicker DTC Remote Consultation With a Non-Referring Physician Rather Than Waiting 20 Days to Consult Their Referring Physician in Person, Depending on the Delay Before the DTC and the Clinical Situation (Weighted Data Set, n=1940)

eTable 5. Factors Associated With Patients' Preferences for a Quicker Direct-to-Consumer Remote Consultation With a Non-Referring Physician Rather Than an In-Person Consultation With Their Referring Physician in 20 Days, (Logistic Regression Model)

eFigure 1. Flow Chart

eFigure 2. Preferences for In-Person Consultation or Remote Modalities With the Referring Physician, Depending on the Clinical Situation (Weighted Data Set, n=1908) of All Patients (Panel A) and of Unserved Patients (Panel B)

This supplemental material has been provided by the authors to give readers additional information about their work.

© 2026 Lenfant T et al. *JAMA Network Open*

eMethods 1: Data collection

The first part of the survey gathered:

1) Demographics

- Age (years old)
- Gender (three possible answers: male, female, I prefer not to answer)
- Geographic zone (zip code)
- Highest diploma ("What is the highest diploma you obtained?" divided into 2 levels: “bachelor’s degree or lower” *versus* “higher than a bachelor's degree”)
- Self-perception of financial situation ("How would you describe your current financial situation?", six possible answers were: 1) Very comfortable, 2) Quite comfortable, 3) Balanced, 4) A little tight and I have to be careful with my finances, 5) It's difficult, 6) I am forced to go into debt to get out of it) put into a binary variable with two levels: Yes (answers 1-4) /No (answers 5-6)

2) Health data

- Long-term conditions: "Which condition(s) do you have from this list?" (You can select multiple answers)
    - High blood pressure
    - Diabetes
    - Thyroid disease or other endocrine disease
    - Pulmonary disease (for example, chronic bronchitis or asthma)
    - Heart disease (for example, myocardial infarction, heart failure...)
    - Kidney disease (for example, chronic kidney failure)
    - Gynecological disease (for example, endometriosis)
    - Gastrointestinal disease (for example, ulcer, inflammatory bowel diseases, liver diseases)
    - Neurological disease (for example, stroke, multiple sclerosis, epilepsy)
    - Rheumatological disease (for example, osteoporosis, arthritis, inflammatory rheumatism...)
    - Cancer or hematological disease (for example, lung cancer, prostate cancer, lymphoma, leukemia)
    - Depression, bipolar disorder, chronic anxiety, or other psychiatric disease
    - Vision problems (for example, glaucoma, cataracts...)
    - Dermatological disease (for example, psoriasis, atopic dermatitis)
    - Infectious disease (for example, HIV infection, tuberculosis...)
    - Autoimmune or autoinflammatory disease (for example, lupus, sarcoidosis, Sjögren's syndrome, ANCA-associated vasculitis)
    - Other(s)
- Self-assessment of anxiety linked to their health, self-assessed by the question "Currently, on a scale from 1 to 6, how much do your health problems worry you?" 1: I am not worried about my health at all; 6: I am extremely worried about my health.
- Self-assessment of Self-management, "On a scale from 1 to 6, how do you consider your management of your condition(s) and treatment(s)? 1: I cannot manage them alone at all; 6: I am completely autonomous in managing my illnesses and treatments.
- Digital Health Care Literacy Scale (scale from 3 to 15, 15 being the highest score) put into a binary variable of two levels (lower DHCLS for 3 to 10, higher DHCLS from 11 to 15)

3) Information about their referring physician

- Specialty (General Practitioner, Medical specialty, Surgical Specialty)
- Length of relationship (less than 5 years, 5 to 10 years, more than 10 years)
- Listening skills evaluated by the patient ("How do you evaluate your physician’s ability to listen to you?" with five possible answers 1) Poor 2) Mediocre 3) Average 4) Good 5) Excellent) put into a binary variable Poor to Average (1-3), Good to Excellent (5-6)

4) Information about their usual consultation settings

- Contact modality most frequently used between in-person, video consultation, phone contacts, and asynchronous message exchanges.
- Availability and Frequency of use of remote modalities

    - Video consultation
    - Phone contacts
    - Asynchronous message exchanges
- Time from departure (from home/work) to the start of an in-person consultation, including transportation, parking, inscription, and waiting room, with three possible answers: 1) less than an hour, 2) one to three hours, 3) more than three hours.
- Delay before the next available in-person appointment with the referring physician, assessed by the question "What is the average delay before an in-person consultation with your doctor (for a non-urgent reason)?" with six possible answers: 1) Less than 1 week, 2) Between 1 and 2 weeks, 3) Between 2 weeks and 1 month, 4) Between 1 and 3 months, 5) Between 3 and 6 months, 6) More than 6 months. Divided into a binary variable "less than two weeks" and "two weeks or more".
- Ability to make personal/professional arrangements to free up time to attend an in-person consultation, assessed by the question "How easy is it for you to free up time to see your doctor?" with four possible answers: 1) It's very easy, I can take a whole day off without any problem. 2) It's easy, I can take half a day off without any problem. 3) It's difficult, I need to make arrangements for family/professional obligations. 4) It's very difficult, almost impossible, to free up time for my appointments. Divided into a binary variable "Able to free up half a day or the entire day" (1-2) and "Difficult to Impossible" (3-4)

© 2026 Lenfant T et al. *JAMA Network Open*

eMethods 2: Pilot phase

The survey was tested in a pilot phase of 30 patients with LTC, recruited through personal contacts. Each patient completed the survey and reviewed each question with the principal investigator (TL) to ensure its clarity, reproducibility, and reliability, as well as the site's functionality and ease of use. The pilot phase led to iterative refinements of the question wording and response options. At the end of the pilot phase, all 30 patients reviewed the final version of the survey.

eMethods 3: Covariates of the logistic regression models

Covariates were patients', physicians', and consultations' characteristics:

i) *Patients' characteristics*: age (<60 vs ≥60), gender, economic difficulties (yes/no), highest diploma (up to bachelor's degree, higher than bachelor's degree), multimorbidity (1 vs ≥2 LTCs), anxiety score (≤4 vs >4), self-management score ("good or excellent" vs "medium or poor"), DHCLS (≤10, >10/15);

ii) *Physicians' characteristics*: physician's listening skills ("good or excellent" vs "medium or poor"), length of relationship (< 5 vs ≥5 years), specialty (general practitioner vs specialist),

iii) *Consultation's characteristics*: location (at hospital vs in the city), easiness to free up time for the consultation (difficult vs easy), time to consultation (<1 vs ≥1 hour), delay to the next available in-person appointment (<2 weeks, ≥2 weeks), use of remote contact ("not available or never used" vs "used"), *iv)* situation (worsening of symptoms, new symptoms, checkup, renewal, and results).

eMethods 4: Number of in-person GP consultations that could be replaced by remote care in France

General practitioners conducted 229 million in-person consultations in 2023 in France and 5.5 million video consultations.[26]

To approximate the proportion of consultations that align with patients with LTCs and our five clinical scenarios (medication renewal, test result discussion, annual check-up, new symptoms, symptom worsening), we referred to preliminary results of the PaRIS study by the DREES.[27] In Graph 6 of the report, the categories “Soins courants pour un problème de longue durée” ("routine care for a long-term condition") and “Soins ponctuels pour un problème de longue durée” ("occasional care for a long-term condition") together account for 58% of consultations. The remaining 42% correspond to consultations not captured by our scenarios, such as acute or urgent problems, preventive care, administrative visits, pediatric or pregnancy-related encounters, and other consultations unrelated to the management of long-term conditions.

In our study, 37% of patients preferred remote care to in-person consultations in their assigned situation.

Applying this proportion to national activity yields the following rough macro-level approximation:

We therefore estimated: *229 million of in-person GP consultations × 58% =*
132.8 million consultations aligned with our situations

132.8 million × 37% = 49.1 million consultations potentially substitutable by remote care

These figures should be interpreted with considerable caution. They are intended solely as an illustrative order-of-magnitude estimate based on stated preferences in standardized scenarios. They do not imply that these consultations should be delivered remotely. Clinical appropriateness, patient–clinician shared decision-making, and contextual factors remain essential determinants of whether remote care is suitable. Furthermore, this approximation may under- or overestimate the true potential, as it does not account for scenarios requiring in-person assessment or other clinical situations that may also be appropriate for remote delivery.

© 2026 Lenfant T et al. *JAMA Network Open*

Characteristic	**RAW**, N = 1,995[1]	**Worsening symptoms**, N = 393[1]	**New symptoms**, N = 412[1]	**Annual checkup**, N = 371[1]	**Result discussion**, N = 358[1]	**Medication renewal**, N = 461[1]
**Age - years (IQR)**	52.0 (40.0, 63.0)	51.0 (38.0, 63.0)	52.0 (40.8, 64.0)	53.0 (41.0, 65.0)	50.0 (38.0, 62.0)	52.0 (40.0, 62.0)
**Gender - no. (%)**						
Male	562 (28%)	127 (32%)	120 (29%)	106 (29%)	88 (25%)	121 (26%)
Female	1,433 (72%)	266 (68%)	292 (71%)	265 (71%)	270 (75%)	340 (74%)
**Highest diploma - no. (%)**						
Higher	1,209 (61%)	248 (63%)	242 (59%)	215 (58%)	219 (61%)	285 (62%)
Lower	786 (39%)	145 (37%)	170 (41%)	156 (42%)	139 (39%)	176 (38%)
**Financial difficulties — no. (%)**						
Economical difficulties	697 (35%)	137 (35%)	149 (36%)	120 (32%)	123 (34%)	168 (36%)
No difficulties	1,298 (65%)	256 (65%)	263 (64%)	251 (68%)	235 (66%)	293 (64%)
**Multimorbidity — no. (%)**	1,323 (66%)	241 (61%)	277 (67%)	257 (69%)	232 (65%)	316 (69%)
**Anxiety — no. (%)**						
4 or lower	1,201 (60%)	239 (61%)	247 (60%)	222 (60%)	216 (60%)	277 (60%)
higher than 4	794 (40%)	154 (39%)	165 (40%)	149 (40%)	142 (40%)	184 (40%)
**Self-management — no. (%)**						
good or excellent	1,248 (63%)	233 (59%)	253 (61%)	231 (62%)	235 (66%)	296 (64%)
medium or poor	747 (37%)	160 (41%)	159 (39%)	140 (38%)	123 (34%)	165 (36%)
**DHCLS — no. (%)**						
higher DHL	1,729 (87%)	342 (87%)	351 (85%)	317 (85%)	319 (89%)	400 (87%)
lower DHL	266 (13%)	51 (13%)	61 (15%)	54 (15%)	39 (11%)	61 (13%)
**Physician's specialty — no. (%)**						
general practicioner	1,259 (63%)	246 (63%)	268 (65%)	212 (57%)	236 (66%)	297 (64%)
specialist	735 (37%)	147 (37%)	144 (35%)	158 (43%)	122 (34%)	164 (36%)
**Length of relationship — no. (%)**						
5 years or more	1,167 (59%)	234 (60%)	256 (62%)	219 (59%)	191 (54%)	267 (58%)
less than 5 years	821 (41%)	158 (40%)	156 (38%)	151 (41%)	163 (46%)	193 (42%)
**Listening skills — no. (%)**						
good or excellent	1,553 (78%)	305 (78%)	334 (81%)	286 (78%)	275 (77%)	353 (77%)
medium or poor	440 (22%)	88 (22%)	78 (19%)	83 (22%)	83 (23%)	108 (23%)
**Travel time to consultation — no. (%)**						
less than an hour	1,195 (60%)	245 (63%)	257 (62%)	209 (56%)	215 (60%)	269 (59%)
more than 1 hour	795 (40%)	147 (38%)	155 (38%)	161 (44%)	143 (40%)	189 (41%)

© 2026 Lenfant T et al. *JAMA Network Open*

Characteristic	**RAW**, N = 1,995[1]	**Worsening symptoms**, N = 393[1]	**New symptoms**, N = 412[1]	**Annual checkup**, N = 371[1]	**Result discussion**, N = 358[1]	**Medication renewal**, N = 461[1]
**Delay before next consultation — no. (%)**						
less than 2 weeks	1,018 (51%)	204 (52%)	217 (53%)	174 (47%)	191 (54%)	232 (50%)
more than 2 weeks	970 (49%)	189 (48%)	193 (47%)	194 (53%)	166 (46%)	228 (50%)
**Easiness to attend in-person — no. (%)**						
difficult	564 (28%)	104 (26%)	117 (28%)	96 (26%)	110 (31%)	137 (30%)
easy	1,429 (72%)	289 (74%)	295 (72%)	274 (74%)	248 (69%)	323 (70%)
**Remote interactions — no. (%)**						
Not available or never used	857 (43%)	162 (41%)	169 (41%)	167 (45%)	169 (47%)	190 (41%)
Used	1,137 (57%)	231 (59%)	243 (59%)	203 (55%)	189 (53%)	271 (59%)
**Elevated Blood Pressure**	439 (22%)	87 (22%)	102 (25%)	87 (23%)	65 (18%)	98 (21%)
**Diabetes**	203 (10%)	38 (9.7%)	39 (9.5%)	47 (13%)	34 (9.5%)	45 (9.8%)
**Thyroid disease**	295 (15%)	61 (16%)	49 (12%)	56 (15%)	58 (16%)	71 (15%)
**Pulmonary disease**	221 (11%)	55 (14%)	40 (9.7%)	41 (11%)	41 (11%)	44 (9.5%)
**Cardiac disease**	179 (9.0%)	38 (9.7%)	34 (8.3%)	35 (9.4%)	28 (7.8%)	44 (9.5%)
**Kidney disease**	108 (5.4%)	21 (5.3%)	24 (5.8%)	25 (6.7%)	15 (4.2%)	23 (5.0%)
**Gynecological disease**	373 (19%)	71 (18%)	78 (19%)	67 (18%)	67 (19%)	90 (20%)
**Digestive disease**	294 (15%)	52 (13%)	54 (13%)	64 (17%)	58 (16%)	66 (14%)
**Neurological disease**	229 (11%)	37 (9.4%)	60 (15%)	44 (12%)	40 (11%)	48 (10%)
**Rheumatological disease**	558 (28%)	100 (25%)	128 (31%)	106 (29%)	107 (30%)	117 (25%)
**Cancer or hematologic**	161 (8.1%)	36 (9.2%)	35 (8.5%)	28 (7.5%)	19 (5.3%)	43 (9.3%)
**Mental health disorder**	530 (27%)	95 (24%)	109 (26%)	90 (24%)	106 (30%)	130 (28%)
**Ophthalmologic disease**	193 (9.7%)	31 (7.9%)	41 (10.%)	48 (13%)	26 (7.3%)	47 (10%)
**Dermatologic disease**	277 (14%)	51 (13%)	52 (13%)	65 (18%)	45 (13%)	64 (14%)
**Infectious disease**	46 (2.3%)	10 (2.5%)	13 (3.2%)	8 (2.2%)	8 (2.2%)	7 (1.5%)
**Autoimmune disease**	233 (12%)	41 (10%)	47 (11%)	55 (15%)	38 (11%)	52 (11%)
**Other**	525 (26%)	87 (22%)	100 (24%)	113 (30%)	100 (28%)	125 (27%)

eTable 1: Patients, physicians and consultations characteristics described by clinical situation (n=1995)

Characteristics		Odd-ratios (95%CI, p)
Age	60 or older	-
	Younger than 60	1.28 (1.00-1.63, p=0.052)
Gender	Female	-
	Male	0.73 (0.57-0.92, p=0.008)
Self-perception of financial situation	Difficult	-
	Not difficult	1.07 (0.86-1.34, p=0.559)
Highest diploma	Higher than a bachelor's degree	-
	Bachelor's degree or lower	0.93 (0.75-1.15, p=0.493)
Multimorbidity (≥2 LTCs)	No	-
	Yes	1.22 (0.99-1.52, p=0.066)
Self-assessed anxiety level (score from 1 to 6, 6 being the highest level of anxiety)	≤ 4	-
	> 4	0.98 (0.78-1.21, p=0.823)
Self-management (score from 1 to 6, 6 being the best self-management)	Good to excellent (4-6)	-
	Medium to poor (1-3)	0.88 (0.71-1.09, p=0.240)
Digital Health Care Literacy Scale (score from 3 to 15, 15 being the highest)	Higher digital literacy (>10)	-
	Lower digital literacy (≤10)	0.51 (0.37-0.70, p<0.001)
Listening skills evaluated by the patient	Good to excellent	-
	Medium to poor	1.35 (1.06-1.73, p=0.016)
Length of relationship with the physician	5 years or more	-
	Less than 5 years	1.01 (0.82-1.23, p=0.949)
Specialty of the physician	General practitioner	-
	Specialist	1.26 (0.92-1.74, p=0.148)
Location of the consultation	At the hospital	-
	In town	0.89 (0.64-1.25, p=0.506)
Ability to make personal/professional arrangements to free up time to attend an in-person consultation	Difficult to Impossible	-
	Ability to free up half a day or the entire day	0.47 (0.37-0.60, p<0.001)
Time from departure to the start of an in-person consultation (transportation, parking, inscription, waiting room)	Less than an hour	-
	More than 1 hour	1.20 (0.97-1.50, p=0.095)
Delay before the next available in-person consultation	Less than 2 weeks	-
	2 weeks or more	1.38 (1.09-1.74, p=0.007)
Availability and Use of Remote contacts (VC, PC, AME)	None available or none used	-
	Used	1.06 (0.86-1.30, p=0.582)
Clinical situation	Medication renewal	-
	Annual checkup	0.27 (0.20-0.37, p<0.001)
	New symptoms	0.28 (0.21-0.38, p<0.001)
	Worsening of symptoms	0.39 (0.29-0.53, p<0.001)
	Discussing results	0.88 (0.65-1.19, p=0.405)

eTable 2: Factors associated with patients' preferences for remote modalities with their referring physician for the follow-up of their long-term conditions (logistic regression model).

Legend: AME: Asynchronous Message Exchanges, LTCs: Long Term Conditions, PC: Phone contacts, VC: Video consultation.

© 2026 Lenfant T et al. *JAMA Network Open*

		Odd-ratios (IC95%, p)
Age	60 or older	-
	Younger than 60	1.09 (0.74-1.60, p=0.674)
Gender	Female	-
	Male	0.58 (0.40-0.84, p=0.004)
Self-perception of financial situation	Difficult	-
	Not difficult	0.96 (0.68-1.36, p=0.829)
Highest diploma	Higher than a bachelor's degree	-
	Bachelor's degree or lower	0.95 (0.69-1.32, p=0.775)
Multimorbidity (≥2 LTCs)	No	-
	Yes	1.21 (0.86-1.69, p=0.275)
Self-assessed anxiety level (score from 1 to 6, 6 being the highest level of anxiety)	≤ 4	-
	> 4	0.93 (0.65-1.32, p=0.668)
Self-management (score from 1 to 6, 6 being the best self-management)	Good to excellent (4-6)	-
	Medium to poor (1-3)	0.92 (0.66-1.28, p=0.608)
Digital Health Care Literacy Scale (score from 3 to 15, 15 being the highest)	Higher digital literacy (>10)	-
	Lower digital literacy (≤10)	0.43 (0.26-0.68, p<0.001)
Listening skills evaluated by the patient	Good to excellent	-
	Medium to poor	1.32 (0.94-1.85, p=0.109)
Length of relationship with the physician	5 years or more	-
	Less than 5 years	1.25 (0.92-1.71, p=0.157)
Specialty of the physician	General practitioner	-
	Specialist	1.25 (0.74-2.11, p=0.395)
Location of the consultation	At the hospital	-
	In town	1.25 (0.72-2.17, p=0.438)
Ability to make personal/professional arrangements to free up time to attend an in-person consultation	Difficult to Impossible	-
	Ability to free up half a day or the entire day	0.41 (0.28-0.59, p<0.001)
Time from departure to the start of an in-person consultation (transportation, parking, inscription, waiting room)	Less than an hour	-
	More than 1 hour	1.30 (0.93-1.82, p=0.119)
Delay before the next available in-person consultation	Less than 2 weeks	-
	2 weeks or more	1.34 (0.92-1.94, p=0.124)
Clinical situation	Medication renewal	-
	Annual checkup	0.46 (0.28-0.73, p=0.001)
	New symptoms	0.30 (0.18-0.48, p<0.001)
	Worsening of symptoms	0.56 (0.35-0.89, p=0.015)
	Discussing results	0.90 (0.57-1.42, p=0.636)

eTable 3: Factors associated with the preference for a remote modality over an in-person consultation with the referring physician among unserved patients (patients with no access to any of the three remote modalities to interact with their physician), logistic regression model.

Legend: LTCs: Long-term Conditions.

	Raw (n=1940)		Weighted (n=1940)		Worsening (n=382)	New (n=405)	Checkup (n=357)	Renewal (n=453)	Results (n=343)
	n	%	n	%	%	%	%	%	%
"Would you like to see an unknown physician sooner remotely (Direct-To-Consumer) rather than you referring physician in 20 days in person ?"									
It depends on the delay	**358**	18,5%	**359**	18,5%	25,6%	28,9%	11,0%	11,5%	14,5%
Yes, I would opt for this DTC appointment	349	18,0%	342	17,6%	26,6%	20,0%	10,7%	16,4%	14,0%
No, I prefer my physician in person in 20 days	1233	63,6%	1239	63,8%	47,8%	51,1%	78,3%	72,1%	71,5%
total answers	1940		1940						

For patients answering they would consider seeing an unknown physician depending on the delay									
If the DTC appointment is in 15-20 days ?									
Total patients asked	358	100%	359	100,0%					
Yes, I would opt for this DTC appointment	33	9,2%	41	11,4%	16,0%	9,8%	11,3%	16,0%	3,4%
No, I prefer my physician in person in 20 days	**322**	89,9%	**316**	88,0%	84,0%	90,2%	88,7%	84,0%	96,6%
missing	*3*	*0,8%*	*2*	*0,6%*					
If the DTC appointment is in 10-15 days ?									
Total patients asked	322	100%	316	100,0%					
Yes, I would opt for this DTC appointment	101	31,4%	103	32,6%	39,3%	30,5%	21,7%	38,7%	31,9%
No, I prefer my physician in person in 20 days	**218**	67,7%	**210**	66,5%	60,7%	69,5%	78,3%	61,3%	68,1%
missing	*3*	*0,9%*	*3*	*0,9%*					
If the DTC appointment is in 5-10 days ?									
Total patients asked	218	100%	210	100,0%					
Yes, I would opt for this DTC appointment	126	57,8%	118	56,2%	70,8%	47,5%	42,1%	53,9%	70,8%
No, I prefer my physician in person in 20 days	**92**	42,2%	**92**	43,8%	29,2%	52,5%	57,9%	46,1%	29,2%
missing	*0*	*0,0%*	0	0,0%					
If the DTC appointment is in the next 5 days ?									
Total patients asked	92	100%	92	100,0%					
Yes, I would opt for this DTC appointment	52	56,5%	45	48,9%	65,8%	56,9%	68,8%	2,8%	33,8%
No, I prefer my physician in person in 20 days	**38**	41,3%	**43**	46,7%	34,2%	43,1%	31,2%	97,2%	66,2%
missing	*2*	*2,2%*	*4*	*4,3%*					

Compared to in person with referring in 20 days, I prefer the unknown physician remotely if the appointment is in …		Weighted (n=1940)	Worsening (n=382)	New (n=405)	Checkup (n=357)	Renewal (n=453)	Results (n=343)
		%	%	%	%	%	%
DTC in a shorter delay without precision	shorter	17,6%	26,6%	20,0%	10,7%	16,4%	14,0%
DTC in a shorter delay + 15-20 days	15-20 d	19,7%	30,7%	22,8%	11,9%	18,2%	14,5%
DTC in a shorter delay + 15-20 d + 10-15 days	10-15 d	25,0%	39,1%	30,8%	14,1%	22,0%	18,9%
DTC in a shorter delay + 15-20 d + 10-15 d + 5-10 days	5-10 d	31,1%	48,4%	39,4%	17,3%	25,2%	25,7%
DTC in a shorter delay + 15-20 d + 10-15 d + 5-10 d + < 5 d	< 5 d	33,4%	50,9%	44,8%	20,3%	25,2%	26,6%

eTable 4: Patients' willingness to trade for a quicker DTC remote consultation with a non-referring physician rather than waiting 20 days to consult their referring physician in person, depending on the delay before the DTC and the clinical situation (weighted data set, n=1940).

© 2026 Lenfant T et al. *JAMA Network Open*

		Odd-ratios (95%CI , p)
Age	60 or older	-
	Younger than 60	0.99 (0.77-1.27, p=0.922)
Gender	Female	-
	Male	1.32 (1.04-1.67, p=0.021)
Self-perception of financial situation	Difficult	-
	Not difficult	0.94 (0.75-1.18, p=0.587)
Highest diploma	Higher than a bachelor's degree	-
	Bachelor's degree or lower	0.87 (0.70-1.08, p=0.219)
Multimorbidity (≥2 LTCs)	No	-
	Yes	0.87 (0.70-1.08, p=0.204)
Self-assessed anxiety level (score from 1 to 6, 6 being the highest level of anxiety)	≤ 4	-
	> 4	0.96 (0.77-1.20, p=0.733)
Self-management (score from 1 to 6, 6 being the best self-management)	Good to excellent (4-6)	-
	Medium to poor (1-3)	0.93 (0.75-1.16, p=0.532)
Digital Health Care Literacy Scale (score from 3 to 15, 15 being the highest)	Higher digital literacy (>10)	-
	Lower digital literacy (≤10)	0.81 (0.59-1.11, p=0.195)
Listening skills evaluated by the patient	Good to excellent	-
	Medium to poor	1.53 (1.20-1.95, p=0.001)
Length of relationship with the physician	5 years or more	-
	Less than 5 years	1.17 (0.95-1.44, p=0.134)
Specialty of the physician	Specialist	-
	General practitioner	1.79 (1.28-2.50, p=0.001)
Location of the consultation	At the hospital	-
	In town	0.72 (0.50-1.02, p=0.068)
Ability to make personal/professional arrangements to free up time to attend an in-person consultation	Ability to free up half a day or the entire day	-
	Difficult to Impossible	1.54 (1.22-1.96, p<0.001)
Time from departure to the start of an in-person consultation (transportation, parking, inscription, waiting room)	Less than an hour	-
	More than 1 hour	1.08 (0.87-1.36, p=0.483)
Delay before the next available in-person consultation	2 weeks or more	-
	Less than 2 weeks	1.43 (1.12-1.82, p=0.003)
Availability and Use of Remote contacts (VC, PC, AME)	Used	-
	None available or none used (unserved)	1.33 (1.09-1.64, p=0.006)
Clinical situation	Medication renewal	-
	Annual checkup	0.75 (0.54-1.06, p=0.103)
	New symptoms	3.16 (2.35-4.26, p<0.001)
	Worsening of symptoms	3.74 (2.77-5.08, p<0.001)
	Discussing results	0.89 (0.64-1.23, p=0.485)

eTable 5: Factors associated with patients' preferences for a quicker Direct-To-Consumer remote consultation with a non-referring physician rather than an in-person consultation with their referring physician in 20 days, (logistic regression model).

Legend: AME: Asynchronous Message Exchanges, DTC: Direct-To-Consumer (remote consultation with a non-referring physician), LTCs: Long Term Conditions, PC: Phone contacts, VC: Video consultation

© 2026 Lenfant T et al. *JAMA Network Open*

eFigure 1: Flow chart

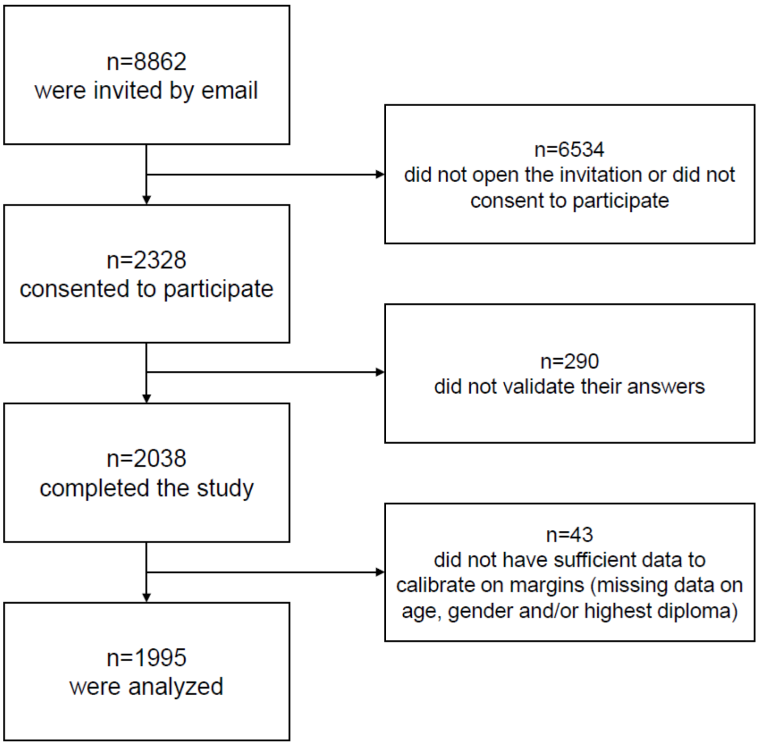

© 2026 Lenfant T et al. *JAMA Network Open*

eFigure 2: Preferences for in-person consultation or remote modalities with the referring physician, depending on the clinical situation (weighted data set, n=1908) of all patients (panel a) and of unserved patients (panel b).

Legend: unserved patients were patients who had no access to or never used any remote modality with their referring physician.

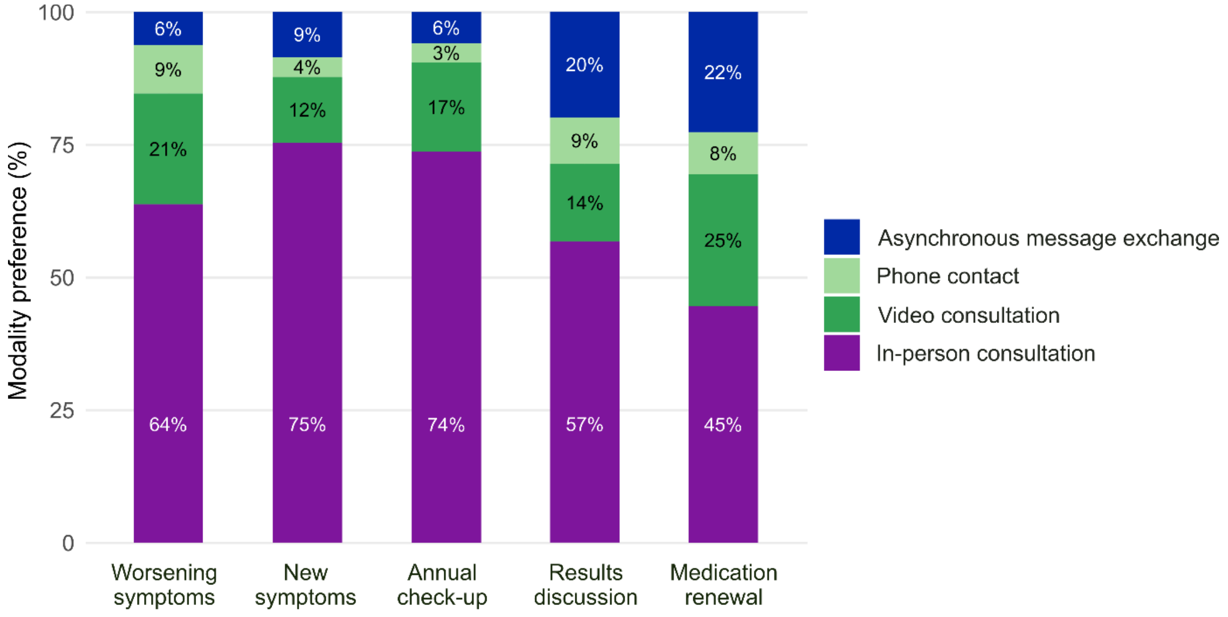

a.

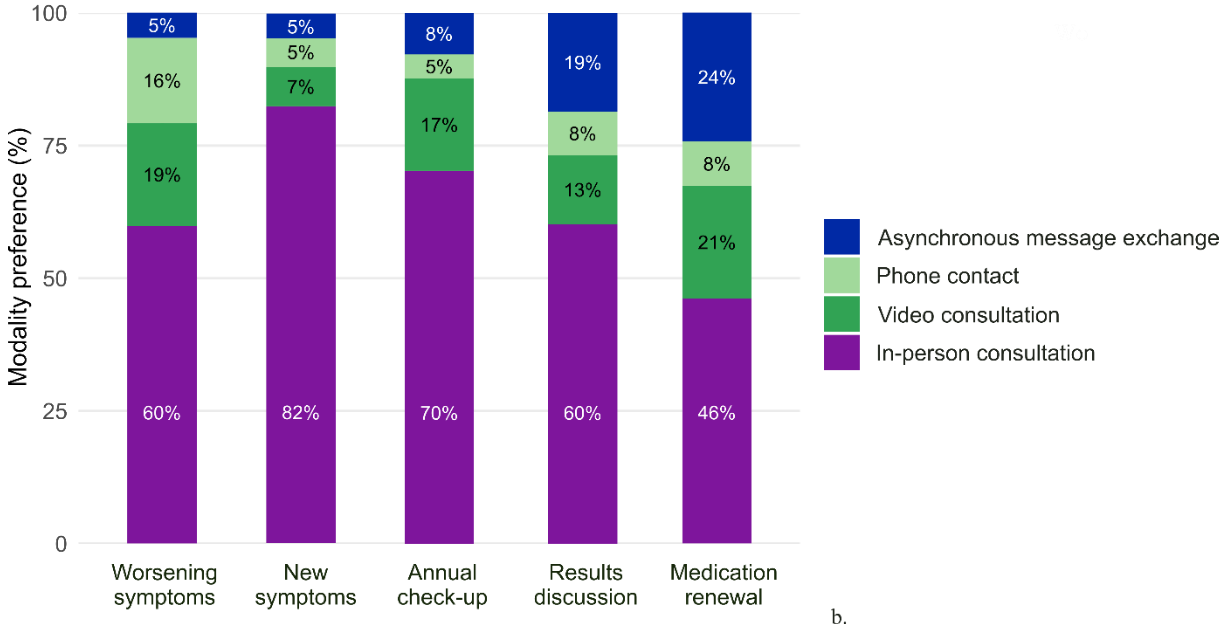

b.

© 2026 Lenfant T et al. *JAMA Network Open*
