# Supplementary material for: Patient Preferences for In-Person vs Remote Care for Long-Term Conditions
Source: JAMA Netw Open. 2026 Feb 10;9(2):e2557759. doi: 10.1001/jamanetworkopen.2025.57759 (PMC12892154; doi:10.1001/jamanetworkopen.2025.57759)
Supplement: Supplement 2. — Data Sharing Statement [file jamanetwopen-e2557759-s002.pdf]

Data Sharing Statement

Lenfant. Patient Preferences for In-Person vs Remote Care for Long-Term Conditions. *JAMA Netw Open*. Published February 10, 2026. doi:10.1001/jamanetworkopen.2025.57759

Data
Data available: No

Additional Information
Explanation for why data not available: The data that support the findings of this study are available from the corresponding author upon reasonable request.
